# Usage patterns, knowledge, and attitudes of healthcare providers regarding e-cigarettes: A cross-sectional study in Saudi Arabia

**DOI:** 10.18332/tid/205871

**Published:** 2025-08-21

**Authors:** Ali M. Alasmari, Ahmed A. Alzahrani, Keir E. J. Philip, Ziyad Alshehri, Saeed M. Alghamdi, Abdullah S. Alsulayyim, Marey A. Almaghrabi, Fahad H. Alahmadi, Abdulrhman M. Hawsawi, Moudi M. Alasmari

**Affiliations:** 1 Respiratory Therapy Department, College of Medical Rehabilitation Sciences, Taibah University, Madinah, Saudi Arabia; 2 National Heart and Lung Institute, Imperial College London, London, United Kingdom; 3 Clinical Technology Department, Respiratory Care Program, College of Applied Medical Science, Umm Al-Qura University, Makkah, Saudi Arabia; 4 Respiratory Therapy Program, Department of Nursing, College of Nursing and Health Sciences, Jazan University, Jazan, Saudi Arabia; 5 Department of Pharmaceutics and Industrial Pharmacy, College of Pharmacy, Taibah University, Madinah, Saudi Arabia; 6 College of Medicine, King Saud bin Abdulaziz University for Health Sciences, Jeddah, Saudi Arabia; 7 King Abdullah International Medical Research Center, Jeddah, Saudi Arabia

**Keywords:** e-cigarettes, healthcare providers, knowledge, attitudes, Saudi Arabia

## Abstract

**INTRODUCTION:**

As electronic cigarettes (e-cigarettes) gain global popularity, healthcare providers (HCPs) play a critical role in shaping public health responses. In Saudi Arabia, little is known about HCPs’ perspectives on e-cigarettes. Hence, this study aimed to evaluate HCPs’ knowledge and attitudes toward e-cigarette use and examine differences based on their personal usage patterns.

**METHODS:**

This is an observational, cross-sectional study. An online questionnaire was distributed from February to May 2024 among HCPs in Saudi Arabia. The survey, which was previously validated, collected data on sociodemographic, smoking characteristics, and 17 items designed to assess HCPs’ knowledge and attitudes about e-cigarette use.

**RESULTS:**

A total of 301 HCPs participated in the study. Among the participants, 19.3% were nurses, 18.9% were PharmDs, 13.2% were dentists, 24.3% were respiratory therapists (RTs), and 24.3% were medical doctors (MDs). Approximately 64% of the respondents were male, and the median age was 32 years (IQR: 22–55). E-cigarette users comprised 22.9% of the respondents. The prevalence of e-cigarette use was highest among dentists (20.0%), with lower rates observed among respiratory therapists (11.0%), nurses (8.6%), pharmacists (7.0%), and medical doctors (6.8%). The majority of respondents (68.1%) recognized that e-cigarettes contain nicotine, 64.5% believed that e-cigarettes are addictive, and 48.9% were unsure whether e-cigarettes are FDA-approved products. Additionally, 33.3% of HCPs relied primarily on social media for information about e-cigarettes. HCPs strongly agreed [median score: 5 (IQR: 4–5)] that HCPs should be educated about e-cigarettes. HCPs who used e-cigarettes exhibited significantly more favorable attitudes toward e-cigarettes compared to non-users, based on the total score (p=0.020).

**CONCLUSIONS:**

HCPs’ knowledge and attitudes regarding e-cigarettes vary widely in Saudi Arabia. Specific, targeted, and regularly updated educational initiatives are needed to ensure that healthcare professionals are confident and well informed regarding the use, risks, and guidelines related to e-cigarettes.

## INTRODUCTION

According to the World Health Organization, the smoking epidemic continues to constitute a significant global health crisis with nearly 8 million people dying annually from smoking-related diseases^1^. The addictive nature of smoking makes it particularly difficult for individuals to quit, with >50% of smokers reporting unsuccessful attempts to stop, despite the health risks^2^. Public health efforts, such as antismoking campaigns and smoking cessation programs, aim to reduce the epidemic’s impact and help individuals overcome nicotine dependence^3^. Electronic cigarettes (e-cigarettes) are claimed to be solutions developed in response to the global tobacco epidemic^4^. Over time, they have also been marketed as safer smoking cessation aids, promising a pathway for smokers to quit traditional tobacco products^5^. In addition, the increasing sales of these cigarettes via online platforms has contributed to the expansion of the e-cigarette market^6^. The Centers for Disease Control and Prevention (CDC) and the US Food and Drug Administration (FDA) reported that 1.63 million (5.9%) middle and high school students currently use e-cigarettes in the United States and that more than 26.3% of them use e-cigarette products daily^7^. In the United Kingdom, 4.7 million (9.1%) adults reported current e-cigarette usage^8^. Moreover, the use of e-cigarettes has been rising in Australia. In 2022–2023, 19.8% of Australians aged ≥14 years reported having used e-cigarettes at least once, while 7.0% reported frequent use. This is increased from 2019 where 11.3% had ever used e-cigarettes and only 2.5% were current users^9^.

Studies conducted in Saudi Arabia and neighboring countries report increasing rates of e-cigarette use among university students and young adults^10^. The main factors contributing to this trend include peer pressure, curiosity, smoking cessation and the perception of reduced harm^11,12^. A research study conducted in Jordan among students from health profession schools revealed that 20% of the students’ used e-cigarettes^13^. In Saudi Arabia, several cross-sectional studies have estimated that the prevalence of e-cigarette use rates ranges from 10% to 30%^14^. A recent study of health college students in Saudi Arabia revealed that the prevalence of e-cigarette use increased to 38.4%, coupled with limited knowledge and misperceptions about e-cigarettes. Additionally, 62% of the students perceived e-cigarettes as stylish alternatives to traditional cigarettes, and more than half of the students agreed that e-cigarettes can cause nicotine dependence^15^.

Although healthcare providers (HCPs) attitudes are a cornerstone in promoting tobacco cessation and in public education about tobacco products’ health hazards, including e-cigarettes, their misconceptions about the health impacts of e-cigarettes still persist. Some studies indicate that many HCPs are either uncertain or misinformed about the risks and benefits of e-cigarettes, whereas others may lack confidence in discussing this emerging public health issue with patients^16,17^. The knowledge and attitudes of healthcare providers, particularly those working with patients who have chronic respiratory diseases, are crucial in guiding public health decisions regarding the use of e-cigarettes. Given the complexity of the public health information surrounding these products, HCPs should serve as primary sources of advice and counselling for smokers seeking cessation^18^.

In Saudi Arabia, the perspectives of HCPs regarding e-cigarettes remain underexplored. Most studies in the Kingdom have focused on the prevalence and determinants of e-cigarette use among the general population, with little attention has been given to healthcare community preparedness to address this emerging trend. This gap is critical, as HCPs are often the first point of contact for patients seeking advice on smoking cessation or the relative safety of alternative tobacco products; without understanding the views and capabilities of HCPs, public health interventions may be ineffective or misaligned with clinical realities. Therefore, this study aims to evaluate HCPs’ knowledge and attitudes toward e-cigarette use in Saudi Arabia, as well as to explore variations in these factors on the basis of personal e-cigarette usage.

## METHODS

### Study design

This is an observational cross-sectional study. An online questionnaire was distributed among HCPs in Saudi Arabia between February and May 2024. The study protocol was granted ethical approval (January 06, 2024) by the Research Ethics Committee of the College of Medical Rehabilitation Sciences, Taibah University, Saudi Arabia (IRB#: CMR-RT-2024-06). All participants gave their electronic informed consent before participating.

### Participants, sampling, and recruitment process

The targeted population for this study was HCPs working in Saudi hospitals. The exclusion criteria include non-healthcare providers or administrative workers. The required sample size was calculated based on estimating the prevalence of e-cigarette use among the Saudi population. Using a previously reported prevalence of e-cigarette use among the Saudi population of 26%^19^, the minimum required sample of 295 participants would be adequate in order to attain 95% confidence interval and 5% margin of error.

The participants were invited via a convenience sampling method and a snowballing technique^20^. First, we approached potential HCPs in Saudi Arabia through publicly available emails or the use of different social media applications, such as the X platform and WhatsApp. The participants were subsequently asked to share the links with their colleagues. Potential participants received an invitation in English describing the study’s purpose and a link to the questionnaire through Google Forms.

### Data collection instrument

The survey instrument was adapted from a validated tool previously used with HCPs in Jordan^21^. The researchers carried out a pilot study to test the final version of their data collection tool. Out of 40 healthcare professionals (HCPs) they approached, 28 participated, 12 were excluded because they were not currently practicing. Feedback from those who took part was used to improve the questionnaire. They conducted reliability test for the tool and reported Cronbach’s alpha scores of 0.677 for the knowledge section and 0.784 for the attitude section, indicating acceptable to good reliability. They also ran a principal component analysis (PCA) to assess sample adequacy. The Kaiser-Meyer-Olkin (KMO) values were 0.679 for knowledge and 0.852 for attitude, both within acceptable ranges, and Bartlett’s test was significant (p<0.001), supporting the tool’s validity.

Modifications were made to facilitate online administration (Supplementary file Table 1). The survey collected data on sociodemographic characteristics (age, gender, marital status, academic level, health profession, and work experience in years), smoking characteristics [smoker or non-smoker, and if a smoker the type (e-cigarettes, traditional, or both), duration, and frequency], and finally, 17 items designed to assess HCPs’ knowledge and attitudes about e-cigarettes. The second section consisted of 17 items designed to assess HCPs’ knowledge about e-cigarettes and their attitudes and was divided into two subscales. The responses to the five knowledge-related items were categorized and reported as ‘no = 0’, ‘not sure=1’, or ‘yes=2’, along with their frequency and percentage (%). The median and interquartile range (IQR) value of the total knowledge scale was calculated, with correct answers coded as 1 and incorrect or ‘not sure’ responses coded as 0, as the latter indicated a lack of knowledge. HCPs’ attitudes were evaluated via 12 items rated on a five-point Likert scale (1=strongly disagree to 5=strongly agree), with higher scores indicating more positive attitudes.

### Data analysis

Data analysis was carried out via SPSS® 27.0 (IBM, Chicago, IL, USA). Continuous variables are presented as the means and standard deviations or medians and interquartile ranges (IQRs), whereas categorical variables are presented as frequencies and percentages. Group comparisons were conducted via the independent sample Mann-Whitney U test. All hypothesis tests were two-sided. A p<0.05 was considered significant.

## RESULTS

### Participant characteristics

In this study, 301 healthcare providers, of whom 64.5% were male, completed the survey. The median age of the participants was 32 years (IQR: 22–55). A total of 51.2% of the respondents were single, 53.5% had over five years of work experience, and 54.2% worked ≤40 hours per week (Table 1).

**Table 1:** Sociodemographic characteristics of HCPs in Saudi Arabia, a cross-sectional study conducted from February to May 2024 (N=301)

*Characteristics*	*n (%)*
**Age** (years), median (IQR)	32 (22–55)
**Gender**	
Female	107 (35.6)
Male	194 (64.5)
**Marital status**	
Single	154 (51.2)
Married	147 (48.8)
**Education level**	
Associate	21 (6.90)
Bachelor’s	180 (59.8)
Master’s	32 (10.6)
PhD/MD/PharmD	68 (22.6)
**Work experience** (years)	
<5	140 (46.5)
5–10	68 (22.6)
>10	93 (30.9)
**Health professions**	
RTs	73 (24.3)
RNs	58 (19.3)
MDs	73 (24.3)
PharmD	57 (18.9)
Dentists	40 (13.3)
**Place of employment**	
Government general hospital	145 (48.2)
Academic hospital	100 (33.2)
Private hospital	32 (10.6)
PHC	24 (7.97)
**Working hours** (per week)	
≤40	163 (54.2)
>40	138 (45.9)

HCPs: healthcare providers. RT: respiratory therapist. RN: registered nurse. MD: medical doctor. PharmD: Doctor of Pharmacy. PHC: primary healthcare.

Among the respondents, 22.9% (n=69) identified themselves as e-cigarette users. Among this group, 75.4% (n=52) reported using e-cigarettes daily, and 78.3% stated that they had been using them for more than a year. Friends were selected as the most common e-cigarette users, with a reported prevalence of 69.8% (Table 2).

**Table 2:** Smoking characteristics of HCPs in Saudi Arabia, a cross-sectional study conducted from February to May 2024 (N=301)

*Characteristics*	*n (%)*
**E-cigarettes and tobacco smoking**	
Non-smokers	214 (71.1)
E-cigarettes alone (no tobacco use)	30 (10.0)
Traditional (tobacco)	18 (5.98)
Dual (e-cigarette and tobacco)	39 (12.9)
**Duration of e-cigarette use** (years) (N=69)	
<1	9 (13.0)
1–3	30 (43.5)
>3	24 (34.8)
Not available	6 (8.7)
**Frequency of e-cigarette use** (N=69)	
Daily	52 (75.4)
Weekly	2 (2.9)
Monthly	11 (15.9)
Not available	4 (5.8)
**Family member uses e-cigarettes**	
No	131 (43.5)
Ex/yes	170 (56.5)
**Friend uses e-cigarettes**	
No	91 (30.2)
Ex/yes	210 (69.8)

### Knowledge about e-cigarettes

As shown in Table 3, 68.1% of HCPs were aware that e-cigarettes contain nicotine, and 64.5% understood the addictive nature of e-cigarettes. Additionally, more than half of the participants (58.0%) considered e-cigarettes to be carcinogenic. Only 37.9% were confident that e-cigarettes are not FDA approved. Overall, half (54.6%) of participants identified correct answers toward attitude. When we categorized HCPs’ responses by their experience of e-cigarette use (users vs non-users), there was no statistically significant difference in total knowledge scores between groups (p=0.083).

**Table 3:** Healthcare providers’ knowledge regarding e-cigarettes in Saudi Arabia, a cross-sectional study conducted from February to May 2024 (N=301)

*Items*	*Yes n (%)*	*No n (%)*	*Not sure n (%)*
E-cigarettes contain nicotine	205 (68.1)^†^	47 (15.6)	49 (16.3)
E-cigarettes considered tobacco products	132 (43.9)^†^	102 (33.9)	67 (22.2)
E-cigarettes carcinogenic	177 (58.8)^†^	79 (26.2)	45 (15.0)
E-cigarettes addictive	194 (64.5)^†^	56 (18.6)	51 (16.9)
E-cigarettes FDA approved for smoking secession	40 (13.2)	114 (37.9)^†^	147 (48.9)

† Correct answers.

**Figure 1:**
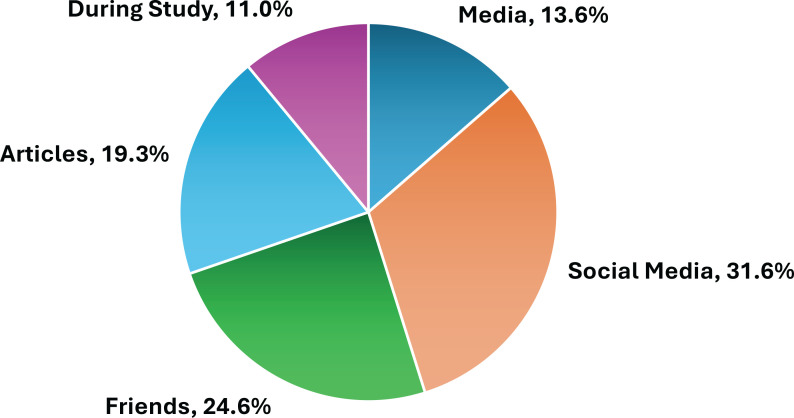
Sources of information about e-cigarettes among HCPs in Saudi Arabia, a cross-sectional study conducted from February to May 2024 (N=301)

Moreover, the participants identified a range of information sources that contributed to their initial understanding of e-cigarettes. Notably, 65.2% relied on less credible channels, such as advertisements, social media, and personal networks (Figure 1).

### Attitudes towards e-cigarettes

With respect to the perceptions and attitudes of HCPs toward e-cigarettes, the median (IQR) of total attitude scale score was 37 (32–43) (Table 4). Among the 12 attitudes items, HCPs believed it important to be educated about e-cigarettes, with the highest positive attitude value of 5 (4–5). This was followed by HCPs’ belief that e-cigarette use should be restricted to public spaces 4 (4–5). Compared with tobacco cigarette use, the perception of HCPs of e-cigarette use can lower the risk of cancer, and patients who are less interested in recommending their patients to use e-cigarettes had the lowest median score of 2 (1–4). Nevertheless, the participants who were ever users of e-cigarettes had shown more favorable attitudes toward e-cigarettes than did the HCPs who were non-users in the total score value (p=0.020).

**Table 4:** Healthcare providers’ attitudes* toward e-cigarettes in Saudi Arabia, a cross-sectional study conducted from February to May 2024 (N=301)

*Items*	*Non-e-cigarette users* *(N=232)* *Median (IQR)*	*E-cigarette users* *(N=69)* *Median (IQR)*	*All* *(N=301)* *Median (IQR)*	*p*
E-cigarette use is safer than smoking tobacco cigarettes	2 (1–4)	4 (2–4)	2 (2–4)	<0.001^a^
E-cigarette vapor is less dangerous than cigarette smoke	2 (2–4)	4 (2–4)	2 (2–4)	<0.001
E-cigarette use is an effective tool for smoking cessation	2 (1–4)	2 (2–4)	2 (1–4)	0.006
E-cigarette use can lower the risk of cancer compared to tobacco cigarettes	2 (1–4)	2 (2–4)	2 (1–4)	0.041
I recommend patients to use e-cigarettes	2 (1–4)	2 (1–4)	2 (1–4)	0.013
It is important to be educated about e-cigarettes	5 (4–5)	5 (4–5)	5 (4–5)	0.874
I feel confident to discuss tobacco cigarette use with my patients	4 (2–5)	4 (2–4.5)	4 (2–5)	0.940
I feel confident to discuss e-cigarette use with my patients	4 (2–4)	4 (2–4)	4 (2–4)	0.518
I received an adequate education about e-cigarettes	2 (2–4)	2 (2–4)	2 (2–4)	0.372
I believe e-cigarette use should be restricted in public spaces	4 (4–5)	4 (2–4.5)	4 (4–5)	<0.001
The cost of e-cigarettes is lower than tobacco products	4 (2–4)	4 (2–5)	4 (2–4)	0.002
I believe e-cigarette use provokes lower public health concerns	4 (2–4)	4 (2–4)	4 (2–4)	0.158
Total attitude	36 (31–42)	39 (34–44.5)	37 (32–43)	0.020

* Attitudes toward e-cigarettes are assessed on a five-point Likert scale (1=strongly disagree to 5=strongly agree). Higher scores indicate more positive attitudes.

a Mann‒Whitney U test. A p<0.05 was considered significant. IQR: interquartile range.

## DISCUSSION

### Main findings

This study suggests that healthcare professionals’ knowledge and attitudes towards e-cigarette use are highly variable in Saudi Arabia. Although about half (52.8%) of participants could correctly identify key knowledge statements, a high proportion could not. Twenty-three percent of the study participants used e-cigarettes, either exclusively or in addition to tobacco products. E-cigarette users demonstrated more positive perceptions of use than non-users, with both groups agreeing that more education for HCPs is needed.

### Significance of findings

This is the first study to explore HCPs’ knowledge and attitudes toward e-cigarette use in Saudi Arabia and assess how variations in these factors are related to personal e-cigarette usage. This is an important topic, given the rapidly increasing prevalence of e-cigarette use in the region and globally. This study provides valuable insights that should prompt specific training and regular updates in this rapidly evolving field. Given the novelty of e-cigarette use and the limited data on health impacts, it is perhaps unsurprising that knowledge and attitudes are variable. This study aligns with, and builds upon, previous related research that has revealed similar variations in the knowledge and attitudes toward e-cigarettes among various healthcare professional groups^22,23^.

Despite the widespread use of e-cigarettes, the majority of the population is unaware that while e-cigarettes can deliver relatively high levels of nicotine, they also contain carcinogenic substances such as formaldehyde, which may lead to serious health issues^24^. Several pathophysiological studies have linked the adverse effects of formaldehyde found in e-cigarettes to increased airway inflammatory markers, oxidative stress, DNA damage, and altered innate host responses, which increase the risk of atherosclerosis and periodontitis^25^. However, scientific evidence about e-cigarettes long-term health effects and efficacy as cessation tools remains inconclusive.

Furthermore, a systematic review of general practitioner knowledge and attitudes regarding the prescription of e-cigarettes as smoking cessation aids revealed a range of positions on this topic, similar to the results of our study^26^. Although some actively promoted their use, others did not recommend e-cigarettes to patients as a smoking cessation aid. Our findings align with those of Australian GPs, showing that those with greater knowledge and a positive attitude were more likely to acknowledge e-cigarettes as effective smoking cessation tools and to recommend them to patients seeking to quit^16^. Studies indicate that many HCPs may lack comprehensive knowledge about e-cigarettes, leading to inconsistent counselling practices^18,27^. For example, a study conducted in the United States reported that 70% of HCPs were unsure about the safety of e-cigarettes and that >50% felt confident in counselling patients about their use^28^. Furthermore, a recently conducted study on HCPs in Jordan revealed that approximately 16.9% of participants were e-cigarette users themselves, and many reported not receiving adequate education regarding e-cigarettes during their professional training^21^. Additionally, another study showed that a considerable proportion of participants incorrectly assumed that e-cigarettes had FDA approval^13^.

A study of nursing students in Croatia reported that more than 50% of study participants used e-cigarettes, which is substantially greater than that reported in our study^29^. They also reported that knowledge and confidence were not particularly high and that education on this topic was perceived to be lacking. Interestingly, they did not find differences in response between smokers and non-smokers. Our findings are also in line with those from Turkey, which indicated that family physicians lacked clinical knowledge about e-cigarettes, affecting their confidence in discussing them with patients, although they expressed interest in formal training^17^. The higher prevalence may be related to the different age groups included in their study and to cultural differences in smoking and e-cigarette use. A 2017 study of physicians on this topic from the United States reported findings similar to ours, with substantial variation in knowledge and practices related to e-cigarettes^22^. These studies, and others, demonstrate that this is a global issue.

With respect to Saudi Arabia, previous studies have explored knowledge and attitudes towards e-cigarette use in specific groups. One survey of medical students revealed substantial variation in perceptions and knowledge, similar to the findings of our study^10^. A study conducted at a Saudi university found that dental students with prior experience using e-cigarettes exhibited greater confidence in their use compared to those without such experience^30^. Alsanea et al.^15^ included students of various health professions and reported widespread misconceptions and a high prevalence of e-cigarette use; interestingly, smokers had lower levels of knowledge than non-smokers.

The prevalence of e-cigarette use in our study was broadly comparable to that reported in other studies. For example, in Jordan, approximately 16.9% of HCPs reported ever e-cigarette users, reflecting a growing trend among professionals as well^21^. Additionally, a study in the United Kingdom reported a prevalence of about 13% of having attempted e-cigarette use among adults, and e-cigarettes were the most popular smoking cessation aid used by 27.2% of smokers trying to quit^31^. The trend is similar in South Korea, where e-cigarette use has been associated with younger age groups and a desire for smoking cessation^32^. In Australia, however, the prevalence of e-cigarette use among adults was lower, at approximately 1.2% in 2019, due to stringent regulations on the sale and marketing of e-cigarettes, such as controlling the availability of nicotine-containing e-cigarettes^33^.

Future research should use a larger sample size and from different geographical locations in Saudi Arabia to be able to generalize findings. Additionally, future studies should consider collecting data from HCPs that work in smoking cessation clinics as they have greater experience in this area and are exposed to patients who smoke.

### Limitations

Certain limitations should be considered when interpreting the findings of this study. First, the cross-sectional design limits the ability to establish causal relationships between knowledge and attitude levels. As with any survey, there is a possibility that responses were influenced by social desirability bias or recall bias. However, this impact may have been mitigated by the anonymization of responses. Second, due to our recruitment methods, individuals with a greater interest in the topic or those more confident in their knowledge may be overrepresented. Additionally, the use of a snowball sampling strategy, which is a form of convenience sampling, can introduce selection bias and may affect the representativeness and generalizability of the results to the broader target population. Furthermore, the small sample size largely due to time constraints during the data collection process makes it difficult to determine how representative our sample is of all HCPs in Saudi Arabia. Consequently, the generalizability of the findings may be limited.

## CONCLUSIONS

Healthcare professionals’ e-cigarette knowledge and attitudes vary widely in Saudi Arabia, which may relate to individuals’ personal use of e-cigarettes. These findings are consistent with similar results reported in the literature, suggesting that this may be a broader issue observed in multiple contexts. Specific, targeted, and regularly updated educational initiatives are needed to ensure that healthcare professionals are confident and well informed regarding the use, risks, and guidelines related to e-cigarettes. These results may influence policymakers in the healthcare sector in Saudi Arabia to initiate continuing professional development training that provides trusted information and resources about e-cigarettes, and be very effective in boosting HCPs’ confidence to discuss them with their patients.

## Supplementary Material



## Data Availability

The data supporting this research are available from the authors on reasonable request.
