# Supplementary material for: Usage patterns, knowledge, and attitudes of healthcare providers regarding e-cigarettes: A cross-sectional study in Saudi Arabia
Source: Tob Induc Dis. 2025 Aug 21;23:10.18332/tid/205871. doi: 10.18332/tid/205871 (PMC12369480; doi:10.18332/tid/205871)
Supplement: Supplementary file 1 [file TID-23-116-s1.pdf]

# Supplement materials:

*Supplementary Table 1. The survey instrument*

| | |
|---|---|
| Participants' demographics: | |
| Age: | …… years |
| Gender | ☐ Female<br>☐ Male |
| Marital status | ☐ Single<br>☐ Married |
| Educational level | ☐ Diploma<br>☐ Bachelor<br>☐ Master<br>☐ PhD/MD/PharmD |
| Work experience | ☐ <5 years<br>☐ 5-10 years<br>☐ >10 years |
| Health profession | ☐ Respiratory therapist<br>☐ Registered nurse<br>☐ Medical doctor<br>☐ PharmD<br>☐ Dentists |
| Place of employment | ☐ Government general hospital<br>☐ Academic hospital<br>☐ Private Hospital<br>☐ Primary Health Care |
| Working hours (per week) | =<40<br>>40 |
| Characteristics practice of e-cigarettes and tobacco smoking: | |
| Non-Smokers | ☐ |
| e-cigarettes-alone (no tobacco use) | ☐ |
| Traditional (Tobacco) | ☐ |
| Dual (e-cigarette and tobacco) | ☐ |
| Duration of e-cigarettes use | ☐ <1 year<br>☐ 1-3 years<br>☐ >3 years |
| Frequency of your e-cigarettes use | ☐ Daily<br>☐ Weekly<br>☐ Monthly |
| Family member uses e-cigarettes | ☐ No<br>☐ Ex/yes |
| Friend uses e-cigarettes | ☐ No<br>☐ Ex/yes |
| What is the main source of your knowledge about e-cigarettes | ☐ Media (TV, radio)<br>☐ Social media platforms (facebook, X, Whattsup)<br>☐ Friends<br>☐ Reading articles<br>☐ During study |
| Participants knowledge and attitude toward e-cigarettes: | |
| Knowledge statements: | |
| e-cigarettes contain nicotine | ☐ Yes<br>☐ No<br>☐ Not sure |
| e-cigarettes considered tobacco products | ☐ Yes<br>☐ No<br>☐ Not sure |
| e-cigarettes carcinogenic | ☐ Yes<br>☐ No<br>☐ Not sure |

| | |
|---|---|
| e-cigarettes addictive | ☐ Yes<br>☐ No<br>☐ Not sure |
| e-cigarettes FDA approved products | ☐ Yes<br>☐ No<br>☐ Not sure |
| Attitude statements: | |
| e-cigarettes use is safer than smoking tobacco cigarettes | ☐ Strongly disagree<br>☐ Disagree<br>☐ Neutral<br>☐ Agree<br>☐ Strongly agree |
| e-cigarettes vapor is less dangerous than cigarette smoke | ☐ Strongly disagree<br>☐ Disagree<br>☐ Neutral<br>☐ Agree<br>☐ Strongly agree |
| e-cigarettes use is an effective tool for smoking cessation | ☐ Strongly disagree<br>☐ Disagree<br>☐ Neutral<br>☐ Agree<br>☐ Strongly agree |
| e-cigarettes use can lower the risk of cancer as compared to tobacco cigarettes | ☐ Strongly disagree<br>☐ Disagree<br>☐ Neutral<br>☐ Agree<br>☐ Strongly agree |
| As a HCP, I recommend patients to use e-cigarettes | ☐ Strongly disagree<br>☐ Disagree<br>☐ Neutral<br>☐ Agree<br>☐ Strongly agree |
| As a HCP, its important to be educated about e-cigarettes | ☐ Strongly disagree<br>☐ Disagree<br>☐ Neutral<br>☐ Agree<br>☐ Strongly agree |
| As a HCP, I feel confident to discuss tobacco cigarettes use with my patients | ☐ Strongly disagree<br>☐ Disagree<br>☐ Neutral<br>☐ Agree<br>☐ Strongly agree |
| As a HCP, I feel confident to discuss e-cigarettes use with my patients | ☐ Strongly disagree<br>☐ Disagree<br>☐ Neutral<br>☐ Agree<br>☐ Strongly agree |
| As a HCP, I received an adequate education about e-cigarettes | ☐ Strongly disagree<br>☐ Disagree<br>☐ Neutral<br>☐ Agree<br>☐ Strongly agree |

| | |
|---|---|
| As a HCP, I believe e-cigarette use should be restricted in public spaces | ☐ Strongly disagree<br>☐ Disagree<br>☐ Neutral<br>☐ Agree<br>☐ Strongly agree |
| The cost of e-cigarettes is lower than tobacco products | ☐ Strongly disagree<br>☐ Disagree<br>☐ Neutral<br>☐ Agree<br>☐ Strongly agree |
| As a HCP, I believe e-cigarettes use provoke lower public health concerns | ☐ Strongly disagree<br>☐ Disagree<br>☐ Neutral<br>☐ Agree<br>☐ Strongly agree |

© 2025 Alasmari A.M. et al.
